# Cancer Incidence Among Users of Glucagon-Like Peptide-1 Receptor Agonists

**DOI:** 10.1007/s11606-026-10300-1

**Published:** 2026-02-26

**Authors:** Zayed Rashid, Selamawit Woldesenbet, Mujtaba Khalil, Abdullah Altaf, Shahzaib Zindani, Areesh Mevawalla, Azza Sarfraz, Khalid Mumtaz, Timothy M. Pawlik

**Affiliations:** 1 Department of Surgery, The Ohio State University Wexner Medical Center and James Comprehensive Cancer Center, Columbus, OH USA; 2 Department of Internal Medicine, Division of Gastroenterology, Hepatology & Nutrition, College of Medicine, The Ohio State University, Columbus, OH USA; 3 Department of Internal Medicine, Conemaugh Memorial Medical Center, Johnstown, PA USA

**Keywords:** GLP-1RA medications, hepatocellular carcinoma, thyroid cancer, pancreas cancer, diabetes, obesity

## Abstract

**Background:**

Glucagon-like peptide-1 receptor agonists (GLP-1RAs) are novel antidiabetic agents that may influence cancer risk. While some studies suggest protective effects, others raise concerns about potential oncogenic associations.

**Objective:**

To investigate the risk of common cancers with GLP-1RA initiation.

**Design:**

Retrospective cohort study.

**Participants and Main Measures:**

Patients diagnosed with type 2 diabetes between 2013–2021 were identified using IBM-MarketScan database and were categorized into exposure (i.e., GLP-1RA) and comparison (i.e., insulin) groups. Overlap Propensity Score Weighting (OPSW), followed by Cox proportional hazards models, was used to assess cancer risk.

**Key Results:**

Among 106,088 patients, most were male (*n* = 44,059, 51.3%), and the mean age was 51 (SD: ± 9.8) years; 50.8% (*n* = 53,924) of patients had GLP-1RA initiation. Overall, 1.9% (*n* = 1,594) of the individuals developed cancer (thyroid: *n* = 110, 0.1%; lung: *n* = 127, 0.1%; breast: *n* = 369, 0.4%; esophagus: *n* = 20, 0.02%; gastric: *n* = 34, 0.03%; liver: *n* = 116, 0.1%; biliary: *n* = 20, 0.02%; pancreatic: *n* = 87, 0.1%; small intestine: *n* = 23, 0.02%; renal: *n* = 128, 0.1%; bladder: *n* = 66, 0.1%; colorectal: *n* = 175, 0.2%; prostate: *n* = 322, 0.4%; ovarian: *n* = 51, 0.1%; endometrial: *n* = 130, 0.2%; neuroendocrine: *n* = 60, 0.1%). Compared to insulin, GLP-1RA medications were associated with a significantly lower risk of liver cancer (HR: 0.47, 95% CI: 0.27–0.82) and pancreatic cancer (HR: 0.23, 95% CI: 0.11–0.51). Risk of all other cancers remained comparable between the two groups (all *p* > 0.05).

**Conclusions:**

GLP-1RA use was associated with a lower incidence of liver and pancreatic cancer, with no increased risk observed for other major cancers. As these medications become more widely used, further research is warranted to better define their long-term cancer-related safety profile.

**Supplementary Information:**

The online version contains supplementary material available at 10.1007/s11606-026-10300-1.

## INTRODUCTION

Cancer is the second leading cause of death in the United States with an estimated increase of over half a million cancer-related deaths annually.^1^ The pathogenesis of most cancer types may differ, but several common modifiable risk factors can be addressed to help prevent many forms of cancer.^2^ These modifiable factors can be environmental (e.g., toxic exposures), behavioral (e.g., alcohol intake), or metabolic (e.g., obesity or diabetes).^2^ Recently, there has been growing emphasis on the investigation of modern therapeutics that can impact cancer incidence either through direct mechanisms or by targeting indirect pathways.^3,4^ One such modern class of medications is glucagon-like peptide-1 receptor agonists (GLP-1 RAs), which were originally approved to manage diabetes and later found to offer additional benefits for obesity and metabolic disorders such as steatohepatitis.^5,6^

GLP-1RA medications primarily work by mimicking endogenous incretins that help regulate key metabolic pathways such as β-oxidation of fatty acids and glycogenolysis by influencing insulin and glucagon secretion.^6^ GLP-1RA medications may also alter molecular pathways such as phosphoinositide 3-kinase/protein kinase B (PI3K/Akt) or cyclic adenosine monophosphate (cAMP) dependent signaling pathways.^7^ Alterations in these molecular signals can impact key pathological processes involved in tumorigenesis such as cellular differentiation, proliferation, migration, and apoptosis.^7^ Of note, a study by Wang et al.^4^ suggested that GLP-1RA medications were associated with a lower colorectal cancer incidence among individuals with diabetes. In contrast, incretin therapy has been linked to an increased risk of thyroid cancer in mouse models, a finding attributed to elevated baseline serum insulin levels.^8^ Similarly, evidence concerning the association between GLP-1RA medications and pancreatic cancer remains conflicting, with some studies indicating an increased risk while others suggest a potential protective effect.^9–11^

Amidst the increasing popularity of GLP-1RA medications for weight loss,^12^ concerns about their potential impact on cancer incidence are growing, yet data related to this possible association remain limited. As such, the objective of the current study was to investigate the association of GLP-1RA medications with the incidence of common thoracic, gastrointestinal, gynecological, and endocrine cancers using a large population of commercially insured individuals.

## MATERIALS AND METHODS

### Database

Individuals who were diagnosed with type 2 diabetes mellitus (T2DM) between 2013 and 2021 were identified from the IBM-MarketScan database. This comprehensive national dataset contains information on 240 million privately insured enrollees and their dependents, early retirees, Consolidated Omnibus Budget Reconciliation Act (COBRA) continuers, and Medicare-eligible retirees with employer-provided Medicare Supplemental plans.^13^ Data on individual-level characteristics of patients are collected across outpatient, inpatient, and prescription drug services. This database has previously been validated to study clinical or epidemiological outcomes.^6,14,15^

### Patient Selection and Exposure

**Figure 1:**
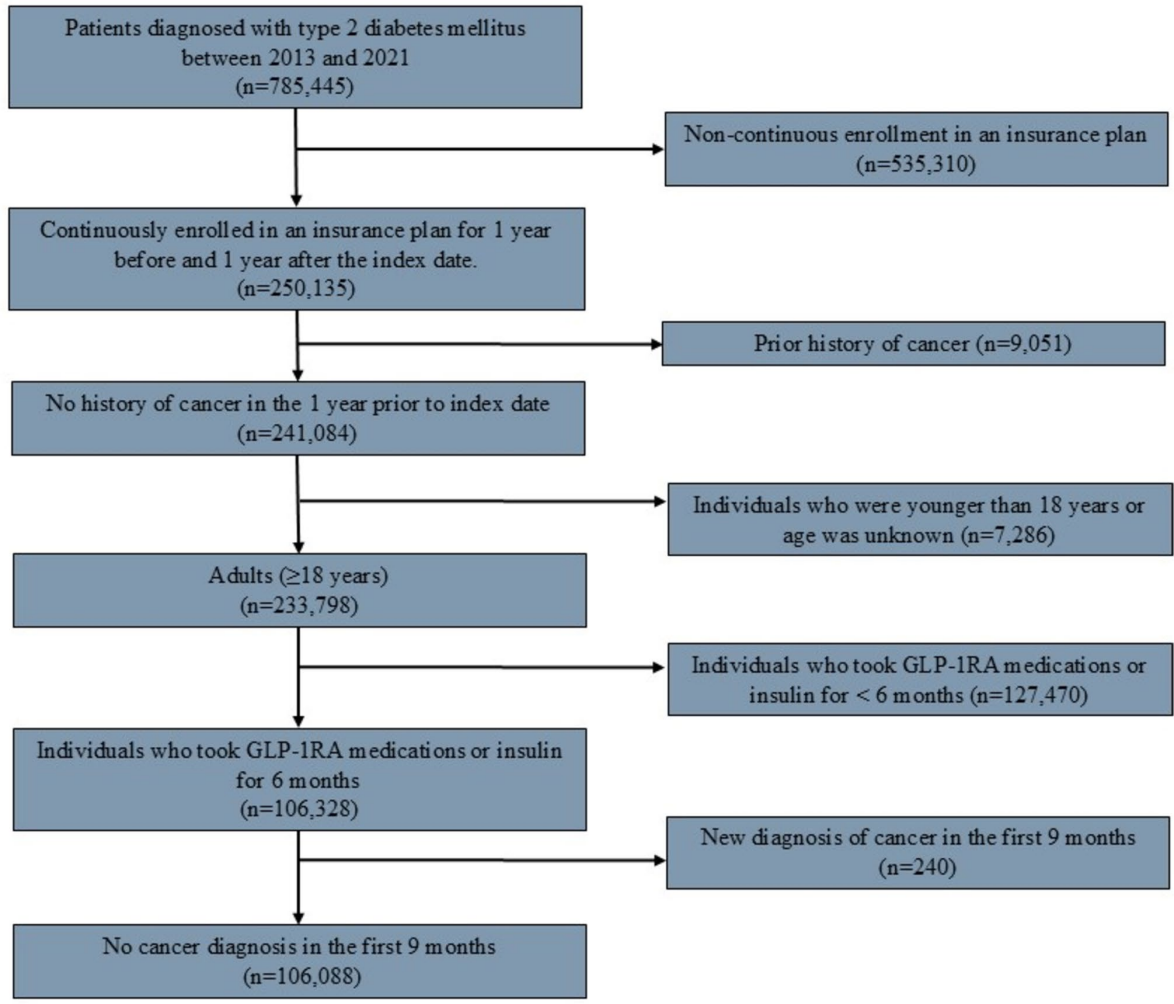
Flowchart depicting the number of patients included according to the study criteria.

Individuals who were 18 to 64 years of age and had at least one inpatient or outpatient diagnosis of T2DM were identified using validated International Classification of Diseases (ICD) ninth and tenth edition codes.^5,6^ Similarly, individuals with continuous enrollment in an insurance plan for one year before and one year after their index diagnosis of T2DM were included. Subsequently, the study cohort was divided into exposure (i.e., patients who took GLP-1RA medications and no insulin) and comparison groups (i.e., patients who took insulin and no GLP-1RA medications). Pharmaceutical records were queried using national drug codes to identify patients with at least one claim for a GLP-1RA medication (Semaglutide, Albiglutide, Liraglutide, Dulaglutide, Lixisenatide, or Exenatide) or insulin after their T2DM diagnosis.^14^ Individuals were followed for six months after the index prescription to ensure continuous exposure to either GLP-1RA medications or insulin.^16^ A subsequent three-month lead-in period was applied before evaluating cancer incidence.^16^ Individuals with prior GLP-1RA use or a cancer diagnosis within one year before or nine months after the index prescription were excluded from the analysis. GLP-1RA *naïvety* was defined as no GLP-1RA medications related pharmacy claim within 365 days preceding the index prescription.^17^ Similarly, individuals aged 18 or younger, as well as individuals of an unknown age were excluded (Fig. 1). The current study adhered to Strengthening the Reporting of Observational Studies in Epidemiology (STROBE) reporting guidelines. The Institutional Review Board at the Ohio State University approved this study; the need for informed consent was waived due to use of de-identified data.

### Variables and outcomes

The variables of interest included patient age, sex, benefit plan type, employment status, region (i.e., Northeast, North Central, South and West), claim-based frailty index, Charlson Comorbidity Index (CCI), rurality, as well as concurrent medical conditions (Table 1). The CCI score was calculated after excluding mild and moderate-to-severe liver disease, as well as diabetes with chronic complications, which were treated as separate variables. Patients were then categorized based on their CCI score, using a cutoff of 2, as previously defined.^18^ Similarly, the claim-based frailty index was estimated from the administrative dataset using a previously validated methodology that incorporates Current Procedural Terminology (CPT) codes, Healthcare Common Procedure Coding System (HCPCS) codes, and ICD codes.^19^ The claim-based frailty index reflects accumulated health deficits during the year preceding the index exposure, and individuals were categorized as non-frail (i.e., < 0.15), prefrail (0.15–0.24), and frail (≥ 0.25). Similarly, patients were classified into three employment status categories: actively employed, retired, and "Other." The "Other" group encompassed dependents, individuals with long-term disabilities, and individuals continuing coverage under COBRA (Consolidated Omnibus Budget Reconciliation Act). Furthermore, ICD-ninth and -tenth edition codes were used to identify baseline comorbidities involving the cardiovascular, central nervous, and gastrointestinal systems (Table 2).^20^

**Table 1:** Baseline Characteristics of Patients With Type 2 Diabetes Mellitus by Initiation of Either Glucagon-like Peptide-1 Receptor Agonist or Insulin

		Unweighted				Weighted ^b^		
Patient characteristics	Total (*N* = 106,088)	GLP-1RA (*N* = 53,924, 50.8)	Insulin (*N* = 52,164, 49.2)	Standard diff. ^a^	p-value	GLP-1RA (%)	Insulin (%)	Standard diff
Age	50.8 ± 9.8	49.3 ± 11.7	52.3 ± 7.9	−30.1	< 0.001	51.8 ± 8.3	51.8 ± 9.8	0
Sex					< 0.001			
Female	41,827 (48.7)	28,318 (52.5)	22,372 (42.9)	−9.6		47.8	47.8	0
Male	44,059 (51.3)	25,606 (47.5)	29,792 (57.1)	−9.6		52.2	52.2	0
Region					< 0.001			
Northeast	19,174 (13.1)	6938 (12.9)	6935 (13.3)	1.2		12.9	12.9	0
North Central	29,674 (20.3)	10,136 (18.8)	11,538 (22.1)	8.2		21.0	21.0	0
South	76,657 (52.5)	31,081 (57.6)	24,502 (47.0)	21.3		52.3	52.3	0
West	18,025 (12.3)	5333 (9.9)	7687 (14.7)	14.7		13.8	13.8	0
Rurality					< 0.001			
Metro	123,906 (84.8)	45,065 (83.6)	44,467 (85.2)	4.4		16.1	15.6	0.005
Nonmetro	22,161 (15.2)	8859 (16.4)	7697 (14.8)	4.4		83.9	84.4	0.005
Claim-based frailty score					< 0.001			
< 0.15	99,573 (68.2)	37,807 (70.1)	35,102 (67.3)	6.0		67.7	67.7	0
0.15–0.25	45,415 (31.1)	15,922 (29.5)	16,509 (31.6)	4.6		31.7	31.7	0
≥ 0.25	1079 (0.7)	195 (0.4)	553 (1.1)	8.1		0.6	0.6	0
CCI					< 0.001			
< 2	103,898 (71.1)	39,571 (73.4)	35,902 (68.8)	10.2		73.4	73.4	0
≥ 2	42,169 (28.9)	14,353 (26.6)	16,262 (31.2)	10.2		26.6	26.6	0
Dyslipidemia	96,388 (66.0)	40,126 (74.4)	30,737 (58.9)	33.3	< 0.001	68.4	68.4	0
Obesity	17,027 (11.7)	8954 (16.6)	3647 (7.0)	30.1	< 0.001	14.0	10.0	0.04
Diabetes with complications	2469 (2.3)	366 (0.7)	2103 (4.0)	21.9	< 0.001	0.8	4.1	0.033
Hypertension	96,895 (66.3)	39,473 (73.2)	31,290 (60.0)	28.3	< 0.001	69.3	69.3	0

GLP1−RA, glucagon−like peptide−1 receptor agonist; PPO, preferred provider organization; HMO, health maintenance organization; POS, point of service; CCI, Charlson comorbidity index

^a^Absolute difference in means/proportions divided by pooled SD. The absolute value greater than 0.10 represented an imbalance between two study groups, smaller values indicate better balance, and a value of zero represented exact balance

^b^Overlap weighted proportions and standardized differences calculated by using all patient demographics and medical history

Additional clinicodemographic factors are outlined in Supplementary Table 1

**Table 2:** Risk of Common Cancers Among Individuals who Took Glucagon-like Peptide-1 Receptor Agonists Compared to Those who Used Insulin

		Unweighted incidence			Incidence rates per 1000 person-years following OPSW (95%CI)		HR (95%CI)^a^
Outcome	Total	GLP-1RA	Insulin	p-value	GLP-1RA	Insulin	
Thyroid cancer	110 (0.1)	54 (0.1)	56 (0.1)	0.160	0.40 (0.30–0.54)	0.49 (0.37–0.66)	0.81 (0.45–1.43)
Lung cancer	127 (0.1)	53 (0.1)	74 (0.2)	0.001	0.50 (0.39–0.66)	0.75 (0.61–0.94)	0.65 (0.40–1.06)
Breast cancer	369 (0.4)	218 (0.5)	151 (0.4)	0.190	3.10 (2.70–3.58)	3.04 (2.58–3.60)	1.01 (0.74–1.38)
Esophagus cancer	20 (0.0)	8 (0.0)	12 (0.0)	0.160	0.10 (0.05–0.19)	0.11 (0.06–0.20)	0.89 (0.27–2.94)
Gastric cancer	34 (0.0)	13 (0.0)	21 (0.1)	0.040	0.12 (0.07–0.22)	0.20 (0.13–0.31)	0.57 (0.21–1.54)
Liver cancer	116 (0.1)	33 (0.1)	83 (0.2)	< 0.001	0.34 (0.24–0.49)	0.71 (0.57–0.90)	0.47 (0.27–0.82) ^b^
Biliary cancer	20 (0.0)	5 (0.0)	15 (0.0)	0.006	0.04 (0.01–0.11)	0.11 (0.06–0.22)	0.32 (0.06–1.59)
Pancreatic cancer	87 (0.1)	22 (0.0)	65 (0.2)	< 0.001	0.14 (0.09–0.23)	0.61 (0.48–0.79)	0.23 (0.11–0.51) ^b^
Small intestinal cancer	23 (0.0)	14 (0.0)	9 (0.0)	0.620	0.11 (0.06–0.20)	0.06 (0.03–0.14)	1.85 (0.46–7.41)
Renal cancer	128 (0.1)	72 (0.2)	56 (0.1)	0.900	0.57 (0.45–0.74)	0.56 (0.43–0.72)	1.01 (0.61–1.67)
Bladder cancer	66 (0.1)	34 (0.1)	32 (0.1)	0.490	0.31 (0.22–0.44)	0.28 (0.20–0.40)	1.05 (0.52–2.12)
Colorectal cancer	175 (0.2)	87 (0.2)	88 (0.2)	0.110	0.71 (0.57–0.88)	0.91 (0.75–1.12)	0.76 (0.50–1.17)
Prostate cancer	322 (0.4)	172 (0.4)	150 (0.4)	0.400	2.85 (2.44–3.36)	2.39 (2.02–2.85)	1.17 (0.85–1.62)
Ovarian cancer	51 (0.1)	23 (0.0)	28 (0.1)	0.130	0.35 (0.24–0.55)	0.63 (0.44–0.93)	0.54 (0.24–1.22)
Endometrial cancer	130 (0.2)	81 (0.2)	49 (0.1)	0.130	1.23 (0.98–1.57)	1.25 (0.97–1.65)	0.97 (0.59–1.59)
Neuroendocrine cancer	60 (0.1)	30 (0.1)	30 (0.1)	0.370	0.24 (0.17–0.36)	0.30 (0.21–0.45)	0.78 (0.38–1.63)

GLP−1RA, glucagon−like peptide−1 receptor agonist

^a^The hazard ratio for cancer was calculated among patients who received GLP−1RA medications compared to those who received insulin

The outcomes of interest included the incidence of thyroid, lung, breast, esophagus, gastric, liver, gall bladder, pancreas, small intestine, colorectal, renal, bladder, prostate, ovarian, endometrial, or neuroendocrine cancer. Individuals were followed until the first occurrence of cancer diagnosis, loss to follow-up, death, up to 5 years after the index exposure or the end of the study period, whichever occurred first. The outcomes were determined by the record of at least one inpatient or two outpatient diagnoses through ICD-9/10 codes.^20^

### Statistical analyses

Descriptive statistics were presented as median values with interquartile ranges for continuous variables and as frequencies and percentages for categorical variables. Categorical variables were compared using either the chi-square or Fisher's exact test (χ2) and continuous variables were compared using either the Wilcoxon rank sum test or Student’s t-test as deemed suitable. The incidence of cancer relative to either the intake of GLP-1RA medications or insulin was studied following Overlap Propensity Score weighting (OPSW) to mitigate the confounding influence of concurrent clinicodemographic characteristics or factors. The OPSW was conducted using roughly 50 variables including patient age, sex, body mass index, adverse social determinants of health, personal or family history of cancer, diabetes-related complications, genetic predisposition to cancer, use of other antidiabetic medications, and weight-loss interventions such as bariatric surgery; a precise balance was achieved (Table 2). OPSW uses logistic regression to estimate propensity scores and ensure covariate balance, closely emulating key aspects of randomized controlled trials such as statistical accuracy and covariate equilibrium.^21^ These features make OPSW more effective than alternative weighting methods, such as Inverse Probability of Treatment Weighting.

Following OPSW, cancer incidence rates per 1,000 person-years were reported, and Cox proportional hazards models were used to compare time-to-event outcomes during the follow-up period. Similarly, cumulative incidences were estimated using the Kaplan–Meier survival analysis after adjusting for baseline clinicodemographic factors. All statistical analyses were performed using SAS 9.4 (SAS Institute, Inc, Cary, NC), hazard ratios (HR) with 95% confidence interval (CI) were reported, and statistical significance was defined as a p-value of less than 0.05.

## RESULTS

Among 106,088 individuals in the cohort, most were male (*n* = 44,059, 51.3%), and mean age was 51 (SD: ± 9.8) years. On the claim-based frailty index, most patients were characterized as non-frail (frailty score: < 0.15) (*n* = 99,573, 68.2%) and prefrail (frailty score: 0.15–0.25) (*n* = 45,415, 31.1%), with a small subset of patients categorized as frail (frailty score: ≥ 0.25) (*n* = 1,079, 0.7%); the majority of patients had a CCI score of < 2 (*n* = 103,898, 71.1%). Among other baseline clinical characteristics, 11.7% (*n* = 17,027) of patients had a diagnosis of obesity, 66.3% (*n* = 96,895) had hypertension. 3.9% (*n* = 5,661) suffered from congestive heart failure, and 5.5% (*n* = 5,882) had a concurrent renal disease. Overall, 50.8% (*n* = 53,924) of patients took GLP-1RA medications. The median duration of GLP-1RA use was 21 months (IQR: 15–31), and the median duration of insulin use was 23 months (IQR: 15–38).

Compared with individuals who took insulin, individuals on GLP-1RA medications were more likely to be female (52.5% vs. 42.9%), and younger (49 vs. 52 years). Similarly, individuals who took GLP-1RA medications versus insulin had lower claim-based frailty scores (i.e., < 0.15: 70.1% vs. 67.3%) and comorbidity scores (i.e., CCI < 2: 73.4% vs. 68.8%) (both *p* < 0.001). Compared with individuals who initiated insulin, patients who took GLP-1RA medications were more likely to have obesity (16.6% vs. 7.0%), dyslipidemia (74.4% vs. 58.9%), hypertension (73.2% vs. 60.0%), or chronic pulmonary disease (12.0% vs. 11.3%) (all *p* < 0.001). Similarly, differences in other key baseline comorbidities were noted. Specifically, patients who took GLP-1RA medications were less likely to have concurrent congestive heart failure (2.4% vs. 5.0%), chronic renal disease (3.5% vs. 7.7%), cerebrovascular disease (2.6% vs. 4.2%), and complicated diabetes (0.7% vs. 4.0%) compared with individuals on insulin (all *p* < 0.001). Individuals on GLP-1RA medications versus insulin were also more likely to use other anti-diabetic medications such as metformin (8.3% vs. 3.7%), sodium-glucose cotransporter-2 inhibitors (7.0% vs. 1.3%), and dipeptidyl peptidase-4 inhibitors (15.3% vs. 10.3%) (all *p* < 0.001) (Table 2). After applying OPSW, all clinicodemographic characteristics of patients in the GLP-1RA versus insulin cohorts were well-balanced with a standard difference of less than 0.1 set as the threshold (Table 1 & Supplementary Table 1).

**Figure 2:**
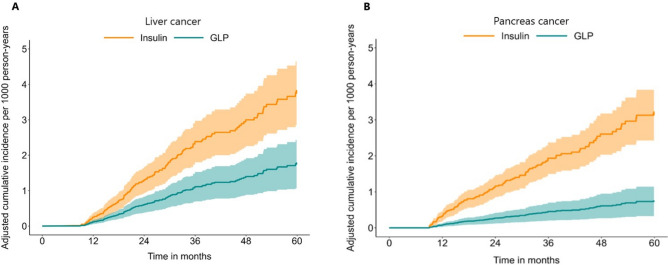
Adjusted cumulative incidence of (**A**) liver cancer and (**B**) pancreas cancer relative to glucagon-like peptide-1 receptor agonist or insulin use.

**Figure 3:**
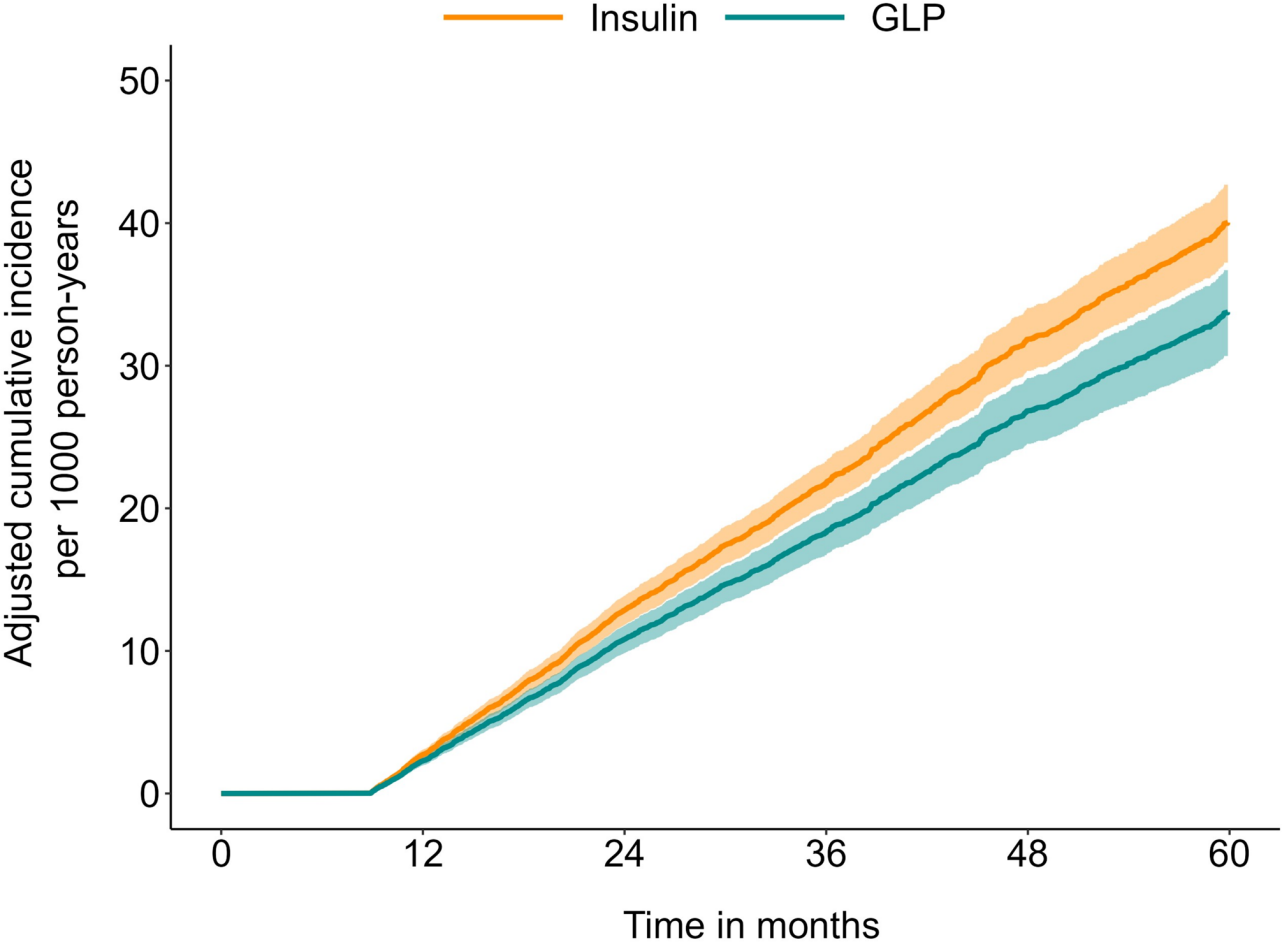
Adjusted cumulative incidence of all cancers combined relative to glucagon-like peptide-1 receptor agonist or insulin use.

Median follow-up time for all patients over the study period was 30 (IQR: 20–46) months (GLP-1RA: 31 (IQR: 20–47) months versus insulin: 30 (IQR: 20–45) months). Overall, 1.9% (*n* = 1,594) of individuals developed cancer (thyroid cancer: *n* = 110, 0.1%; lung cancer: *n* = 127, 0.1%; breast cancer: *n* = 369, 0.4%; esophagus cancer: *n* = 20, 0.02%, gastric cancer: *n* = 34, 0.03%; liver cancer: *n* = 116, 0.1%; biliary cancer: *n* = 20, 0.02%; pancreatic cancer: *n* = 87, 0.1%; small intestine cancer: *n* = 23, 0.02%; renal cancer: *n* = 128, 0.1%; bladder cancer: *n* = 66, 0.1%; colorectal cancer: *n* = 175, 0.2%; prostate cancer: *n* = 322, 0.4%; ovarian cancer: *n* = 51, 0.1%; endometrial cancer: *n* = 130, 0.2%; neuroendocrine cancer: *n* = 60, 0.1%). Of note, the incidence of all cancer types combined was lower among individuals taking GLP-1RA medications (6.5%, 95%CI 6.1–7.0) versus insulin (7.7%, 95%CI 7.2–8.2). When stratified by cancer-type, patients on GLP-1RA had a lower incidence per 1000 person-years for both liver cancer (GLP-1RA medications: 0.3%, 95%CI 0.2–0.5 vs. insulin: 0.7%, 95%CI 0.6–0.9) and pancreatic cancer (GLP-1RA medications: 0.1%, 95%CI 0.1–0.2 vs. insulin: 0.6%, 95%CI 0.5–0.8). In contrast, the incidence of other cancer types was similar among individuals taking GLP-1RA medications or insulin (Table 2). The Cox proportional hazard models demonstrated that GLP-1RA medications were associated with a lower risk of developing liver (HR: 0.47, 95%CI 0.27–0.82) and pancreatic cancer (HR: 0.23, 95%CI 0.11–0.51) compared with patients on insulin (both *p* < 0.05) (Fig. 2A and B). In contrast, the risk of all other cancers was comparable among individuals who used GLP-1RA medications versus insulin (Table 2) (Fig. 3). A sensitivity analysis based on patient sex was performed to account for sex-specific cancers, which demonstrated consistent findings. Similarly, sensitivity analysis was performed after excluding patients who took DPP-4 inhibitors and results were consistent (Supplementary Table 2).

## DISCUSSION

Around 10 million people die from cancer each year worldwide, and this number is projected to double by 2050.^22^ Amidst the rising global cancer burden, increasing attention is being directed toward modern therapeutics with the potential to influence tumorigenesis.^3,4^ Of note, a newer class of antidiabetic medications known as GLP-1 receptor agonists can influence both metabolic, as well as molecular pathways.^5^ The complex interplay of these factors can ultimately reshape the cellular microenvironment and modulate carcinogenesis.^7^ In fact, previous studies have reported conflicting evidence regarding the impact of these novel medications on cancer incidence.^9,11,20^ Some studies have suggested that GLP-1RA medications may reduce the incidence of gastrointestinal cancers,^4^ such as colorectal cancer; however, other reports raised concerns about a potential increase in thyroid cancer risk.^23^ As such, the current study was important as it utilized a large cohort of commercially insured individuals to examine the impact of GLP-1RA medications on the incidence of common thoracic, gastrointestinal, gynecologic, and endocrine cancers. Of note, compared with individuals taking insulin, patients on GLP-1RA medications had a lower risk of liver and pancreas cancer. In contrast, the risk of thyroid and neuroendocrine cancer was comparable among patients who did versus did not take GLP-1RA medications. With the widespread popularity of GLP-1 receptor agonists among both younger and older populations, findings of the current study highlight the need to better define the cancer-associated implications of these weight-loss diabetic medications.

GLP-1RA medications have demonstrated promising results to manage common morbidities such as diabetes, obesity, hypercholesterolemia, and hypertension.^24^ In turn, these medications may preferentially improve liver outcomes among patients with underlying liver conditions such as metabolic-associated steatohepatitis or alcohol associated liver disease.^5,6^ Interestingly, in vitro studies of liver cancer cells have demonstrated that GLP-1RA medications can have a direct effect on hepatocellular carcinoma (HCC) through the regulation of tumor suppressor or proto-oncogenes and induce cell cycle arrest, autophagy, and senescence in cancer cells.^25^ In fact, Lu et al. demonstrated that GLP-1RA medication can enhance natural killer cell-mediated cytotoxicity against HCC through suppression of interleukin-6/signal transducer and activator of transcription 3 (IL-6/STAT3) signaling pathway.^26^ In a separate study, Kojima et al. demonstrated that GLP-1RA drugs prevented the progression of HCC among mice models with non-alcoholic steatohepatitis.^27^ Interestingly, the current study noted that individuals using GLP-1 receptor agonists had a 53% (HR: 0.47) lower risk of developing liver cancer versus individuals treated with insulin. These results align with the findings of Wang et al.,^28^ who reported a reduced incidence of liver cancer among individuals treated with GLP-1 receptor agonists. Of note, one of the key protective mechanisms of GLP-1RA medications against cancer is lipolysis, which helps reduce tumorigenic adipokines such as leptin and promotes tumor-suppressive factors such as adiponectin.^29^ The current study demonstrated that individuals with obesity more frequently used GLP-1RA medications compared with insulin (14.0% vs. 10.0%), which may have contributed to observed reduced risk of liver cancer. Collectively, the data suggest that the GLP-1 receptor class of medications may have benefits beyond diabetes or weight management with direct implications for liver cancer risk.

There are some organs such as the pancreas where GLP-1 receptors are abundantly expressed and can have complex interactions with GLP-1RA medications.^30^ Of note, there are theoretical concerns that sustained pharmacological stimulation of these receptors can induce pancreatic ductal cell proliferation or hyperplasia.^31^ These concerns are supported by preclinical work by Perfetti et al. in which incretins were associated with an expansion of pancreatic ducts and glands among rodents.^31^ Similarly, GLP-1RA medications, compared to other traditional weight-loss medications, were found to be associated with the risk of pancreatic inflammation.^32^ These combined mechanistic factors may predispose individuals using GLP-1RA medications to pancreatic cancer. Nonetheless, observational studies have not demonstrated any association of incretin therapy with an increased risk of pancreatic cancer.^10^ In fact, Ayoub et al. noted that GLP-1RA medications may help protect against the development of pancreatic cancer.^33^ The findings of the current study also support the notion that GLP-1RA initiation may be associated with reduced risk of pancreatic cancer. This phenomenon can be explained by the inhibition of PI3K/Akt signaling pathways involved in cellular survival and proliferation by GLP-1 receptor agonists.^34^ Furthermore, unlike normal pancreatic cells, pancreatic cancer cells generally demonstrate reduced or absent expression of GLP-1 receptors.^34,35^ This finding is further supported by experimental studies in both cell culture models and mouse models in which GLP-1 receptor agonists were noted to promote cellular differentiation.^34^ In turn, the relationship between GLP-1RA medications and pancreatic cancer involves multifactorial and complex processes that require further investigation.

GLP-1RA medications have been suggested to increase the risk of other malignancies such as thyroid cancer.^23^ These concerns were, however, largely based on the preclinical data from animal studies; however, clinical evidence of thyroid cancer among humans remains limited. Interestingly, some data have suggested that the effect of GLP-1RA on thyroid cancer may not pertain to humans.^36^ Despite limited evidence, the federal drug authority (FDA) has recommended avoidance of GLP-1RA medication use among individuals who have any history of familial thyroid cancer or genetic predisposition to multiple endocrine neoplasia.^37^ As a result, patients with these risk factors are often excluded from being prescribed GLP-1RA medications to improve glycemic control. Of note, the current study noted a comparable incidence of thyroid cancer among patients who did versus did not use GLP-1RA medications. These findings may reflect that physicians were already avoiding GLP-1RA medications based on FDA recommendations. In turn, there is an ongoing need to reassess the possible association of GLP-1RA medications and thyroid cancer risk. The FDA had issued boxed warnings for other medications based on preclinical data that were later contradicted by large-scale clinical trials.^38,39^ For example, teriparatide had a boxed warning of the incidence of osteosarcoma based on rat models.^39^ Long-term human data did not support this claim, however, and the boxed warning was later removed.^39^

The findings of this study should be interpreted in light of several limitations. Retrospective administrative databases such as MarketScan are subject to possible coding misclassification bias. As the database included only individuals with employer-sponsored benefit plans, the findings may not be generalizable to uninsured populations or individuals covered by government programs such as Medicare or Medicaid.^13^ The retrospective observational design also limited the ability to prove causality between GLP-1RA use and cancer incidence. The use of a causal inference method (i.e., overlap propensity score weighting) helped mitigate confounding by indication. Moreover, information on certain patient-level factors (e.g., race/ethnicity) and granular clinical details (e.g., HbA1c levels), which may influence the observed associations, was lacking. Similarly, variability in modes of administration and dosages limited the ability to reliably analyze dosing of GLP-1RA medications and insulin. Despite these limitations, the current study offered important insights into the association between GLP-1RA medications and the incidence of various cancer types in a large, nationally representative cohort of commercially insured individuals.

In conclusion, among commercially insured individuals, GLP-1RA medications were associated with a lower risk of liver and pancreatic cancers compared with insulin use. GLP-1RA medications did not impact the risk of other common cancers, including thyroid cancer, compared with individuals using insulin. These findings suggest that GLP-1RAs may offer additional oncologic benefits for certain cancers beyond their established metabolic effects. Given the growing use of these medications, especially for weight loss, further prospective studies and clinical trials are warranted to better define their long-term cancer-related safety profile. Personalized risk stratification tools may also help guide safer and more individualized use of GLP-1RA therapy in clinical practice.

## Supplementary Information

Below is the link to the electronic supplementary material.

Supplementary file1 (DOCX 29 KB)

## Data Availability

The data for this study were obtained from the IBM Marketscan database. There are restrictions to the availability of this data, which is used under license for this study. Data can be accessed with permission from the IBM Marketscan Commercial Database.
