# Supplementary material for: Cancer Incidence Among Users of Glucagon-Like Peptide-1 Receptor Agonists
Source: J Gen Intern Med. 2026 Feb 26;41(7):1771–9. doi: 10.1007/s11606-026-10300-1 (PMC13176436; doi:10.1007/s11606-026-10300-1)
Supplement: Supplementary file 1 — Supplementary file1 (DOCX 29 KB) [file 11606_2026_10300_MOESM1_ESM.docx]

| **Supplementary Table 1**. Additional clinicodemographic characteristics of patients with type 2 diabetes mellitus by initiation of either glucagon-like peptide-1 receptor agonist or insulin | | | | | | | | |
| --- | --- | --- | --- | --- | --- | --- | --- | --- |
|  |  | Unweighted | | | | Weighted | | |
| Patient characteristics | Total (N=106,088) | GLP-1RA (N=53,924, 50.8) | Insulin (N=52,164, 49.2) | Standard diff.^a^ | p-value | GLP-1RA (%) | Insulin (%) | Standard diff. |
| Type of health insurance |  |  |  |  | <0.001 |  |  |  |
| PPO | 83,651 (57.3) | 30,270 (56.1) | 30,142 (57.8) | 3.4 |  | 56.4 | 56.4 | 0 |
| HMO | 16,568 (11.3) | 5774 (10.7) | 6434 (12.3) | 5.0 |  | 11.7 | 11.7 | 0 |
| Comprehensive | 5457 (3.7) | 1765 (3.3) | 1988 (3.8) | 2.7 |  | 3.7 | 3.7 | 0 |
| POS | 10,832 (7.4) | 3725 (6.9) | 4082 (7.8) | 3.4 |  | 7.6 | 7.8 | 0 |
| Other | 29,559 (20.2) | 12,390 (23.0) | 9518 (18.2) | 11.9 |  | 20.6 | 20.3 | 0.003 |
| Employment status |  |  |  |  | <0.001 |  |  |  |
| Full time | 97,643 (66.8) | 37,688 (69.9) | 32,491 (62.3) | 16.1 |  | 65.4 | 65.4 | 0 |
| Retiree | 13,224 (9.1) | 4775 (8.9) | 4818 (9.2) | 1.0 |  | 9.6 | 9.6 | 0 |
| Other | 35,200 (24.1) | 11,461 (21.3) | 14,855 (28.5) | 16.7 |  | 25.1 | 25.1 | 0 |
| AIDS | 463 (0.3) | 154 (0.3) | 164 (0.3) | 0.0 | 0.391 | 0.3 | 0.3 | 0 |
| Congestive heart failure | 5661 (3.9) | 1292 (2.4) | 2609 (5.0) | 13.8 | <0.001 | 3.6 | 3.6 | 0 |
| Chronic pulmonary disease | 17,050 (11.7) | 6450 (12.0) | 5881 (11.3) | 2.2 | <0.001 | 1.8 | 1.8 | 0 |
| Dementia | 109 (0.1) | 31 (0.1) | 78 (0.1) | 0.0 | <0.001 | 0.1 | 0.1 | -0.001 |
| Any malignancy ^c^ | 1219 (1.1) | 486 (0.9) | 733 (1.4) | 4.7 | <0.001 | 1.2 | 1.2 | 0 |
| Paralysis | 504 (0.5) | 103 (0.2) | 401 (0.8) | 8.5 | <0.001 | 0.2 | 0.8 | -0.005 |
| Chronic renal disease | 5882 (5.5) | 1865 (3.5) | 4017 (7.7) | 18.3 | <0.001 | 5.1 | 5.1 | 0 |
| Rheumatic disease | 1847 (1.7) | 906 (1.7) | 941 (1.8) | 0.8 | 0.123 | 1.8 | 1.8 | 0 |
| Solid tumor | 140 (0.1) | 34 (0.1) | 106 (0.2) | 2.6 | <0.001 | 0.1 | 0.2 | 0.001 |
| Peptic ulcer disease | 365 (0.3) | 207 (0.4) | 158 (0.3) | 1.7 | 0.024 | 0.4 | 0.4 | 0 |
| Cerebrovascular disease | 3571 (3.4) | 1384 (2.6) | 2187 (4.2) | 8.8 | <0.001 | 3.4 | 3.4 | 0 |
| Peripheral vascular disease | 2719 (2.6) | 1031 (1.9) | 1688 (3.2) | 8.3 | <0.001 | 2.5 | 2.5 | 0 |
| Mild Liver Disease | 6988 (6.6) | 3886 (7.2) | 3102 (5.9) | 5.3 | <0.001 | 6.8 | 6.8 | 0 |
| Severe Liver Disease | 403 (0.4) | 64 (0.1) | 339 (0.6) | 8.5 | <0.001 | 0.2 | 0.2 | 0 |
| Myocardial infarction | 1764 (1.7) | 446 (0.8) | 1318 (2.5) | 13.4 | <0.001 | 1.4 | 1.4 | 0 |
| NAFLD | 5859 (5.5) | 3639 (6.7) | 2220 (4.3) | 10.5 | <0.001 | 5.8 | 5.8 | 0 |
| Alcohol-associated liver disease | 1275 (1.2) | 425 (0.8) | 850 (1.6) | 7.4 | <0.001 | 1.2 | 1.2 | 0 |
| Alpha glucosidase deficiency | 80 (0.1) | 35 (0.1) | 45 (0.1) | 0.0 | 0.205 | 0.1 | 0.1 | 0 |
| Sulphonylureas | 7366 (6.9) | 3571 (6.6) | 3795 (7.3) | 2.8 | <0.001 | 8.2 | 8.2 | 0 |
| Metformin | 6404 (6.0) | 4469 (8.3) | 1935 (3.7) | 19.5 | <0.001 | 5.8 | 5.8 | 0 |
| statin | 4373 (4.1) | 2423 (4.5) | 1950 (3.7) | 4.0 | <0.001 | 4.1 | 4.1 | 0 |
| DPP4 | 13,644 (12.9) | 8275 (15.3) | 5369 (10.3) | 15.0 | <0.001 | 14.4 | 14.4 | 0 |
| SGLT2 inhibitors | 4468 (4.2) | 3769 (7.0) | 699 (1.3) | 28.9 | <0.001 | 3.0 | 3.0 | 0 |
| Thiazolidinediones | 1747 (1.6) | 1337 (2.5) | 410 (0.8) | 13.4 | <0.001 | 1.6 | 1.6 | 0 |
| Adverse SDOH | 263 (0.2) | 164 (0.3) | 99 (0.2) | 2.0 | <0.001 | 0.3 | 0.2 | 0 |
| Family History of cancer | 1933 (1.8) | 1326 (2.5) | 607 (1.2) | 9.7 | <0.001 | 1.6 | 1.6 | 0 |
| Personal history of cancer | 572 (0.5) | 326 (0.6) | 246 (0.5) | 1.4 | 0.003 | 0.6 | 0.6 | 0 |
| Family history of cancer of digestive organ | 1192 (1.1) | 733 (1.4) | 459 (0.9) | 4.7 | <0.001 | 1.1 | 1.1 | 0 |
| Family history of colon polyp | 353 (0.3) | 227 (0.4) | 126 (0.2) | 3.7 | <0.001 | 0.3 | 0.3 | 0 |
| Genetic susceptibility | 48 (0.0) | 36 (0.1) | 12 (0.0) | 4.5 | 0.001 | 0.1 | 0.0 | 0 |
| Nicotine use disorder | 8102 (7.6%) | 3697 (6.9) | 4405 (8.4) | 5.6 | <0.001 | 7.9 | 7.9 | 0 |
| Crohn’s disease | 177 (0.2%) | 46 (0.1) | 131 (0.3) | 4.5 | <0.001 | 0.1 | 0.2 | 0 |
| Ulcerative colitis | 219 (0.2%) | 58 (0.1) | 161 (0.3) | 4.5 | <0.001 | 0.2 | 0.2 | 0 |
| Cystic fibrosis | 125 (0.1%) | 11 (0.0) | 114 (0.2) | 6.3 | <0.001 | 0.0 | 0.0 | 0 |
| Colon polyps | 3742 (3.5%) | 2483 (4.6) | 1259 (2.4) | 12.0 | <0.001 | 4.6 | 4.3 | 0 |
| Benign neoplasm of colon and rectum | 4622 (4.4%) | 2679 (5.0) | 1943 (3.7) | 6.4 | <0.001 | 4.6 | 4.3 | 0.002 |
| Encounter for cancer screening | 7464 (7.0%) | 5400 (10.0) | 2064 (4.0) | 23.7 | <0.001 | 6.9 | 6.9 | 0 |
| Colonoscopy | 119 (0.1%) | 25 (0.0) | 94 (0.2) | 6.3 | <0.001 | 0.1 | 0.1 | 0 |
| Bariatric surgery | 1706 (1.6%) | 819 (1.5) | 887 (1.7) | 1.6 | 0.019 | 1.4 | 1.4 | 0.002 |
| Alcohol use disorder | 1096 (1.0%) | 274 (0.5) | 822 (1.6) | 10.8 | <0.001 | 0.8 | 0.8 | 0.008 |
| BMI 20-24.9 | 733 (0.7%) | 170 (0.3) | 563 (1.1) | 9.6 | <0.001 | 0.3 | 1.2 | 0.01 |
| BMI 25-25.9 | 430 (0.4%) | 188 (0.3) | 242 (0.5) | 3.2 | 0.003 | 0.4 | 0.4 | 0 |
| BMI 26-26.9 | 536 (0.5%) | 259 (0.5) | 277 (0.5) | 0.0 | 0.244 | 0.5 | 0.5 | 0 |
| BMI 27-27.9 | 669 (0.6%) | 347 (0.6) | 322 (0.6) | 0.0 | 0.590 | 0.7 | 0.7 | 0 |
| BMI 28-28.9 | 764 (0.7%) | 433 (0.8) | 331 (0.6) | 2.4 | 0.001 | 0.8 | 0.8 | 0 |
| BMI 29-29.9 | 911 (0.9%) | 558 (1.0) | 353 (0.7) | 3.3 | <0.001 | 0.9 | 0.9 | 0 |
| BMI 30-39 | 8801 (8.3%) | 6319 (11.7) | 2482 (4.8) | 25.3 | <0.001 | 7.8 | 7.8 | 0 |
| BMI 40 or more | 6580 (6.2%) | 4760 (8.8) | 1820 (3.5) | 22.2 | <0.001 | 5.7 | 5.7 | 0 |
| ^GLP1-RA, glucagon-like peptide-1 receptor agonist; AIDS, acquired immunodeficiency syndrome; NAFLD, non-alcoholic fatty liver disease; BMI, body mass index; SDOH, social determinants of health; SGLT-2, sodium-glucose cotransporter-2; DPP-4, dipeptidyl peptidase-4 inhibitors; a Absolute difference in means/proportions divided by pooled SD. The absolute value greater than 0.10 represented an imbalance between two study groups; smaller values indicate better balance, and a value of zero represented exact balance. b Overlap weighted proportions and standardized differences calculated by using all patient demographics and medical history. c Include previous history of any malignancy other than those studied as outcomes.^ | | | | | | | | |

| **Supplementary Table 2:** Risk of common cancers among individuals who took glucagon-like peptide-1 receptor agonists compared to those who used insulin after excluding patients who took dipeptidyl peptidase-4 inhibitors | |
| --- | --- |
|  | HR (95%CI) ^a^ |
| Outcome |  |
| Thyroid cancer | 0.74 (0.40-1.36) |
| Lung cancer | 0.65 (0.39-1.09) |
| Breast cancer | 1.04 (0.76-1.44) |
| Esophagus cancer | 0.92 (0.26-3.22) |
| Gastric cancer | 0.54 (0.19-1.53) |
| Liver cancer | 0.44 (0.25-0.81) |
| Biliary cancer | 0.22 (0.03-1.52) |
| Pancreatic cancer | 0.20 (0.08-0.47) |
| Small intestinal cancer | 1.94 (0.46-8.21) |
| Renal cancer | 1.06 (0.62-1.79) |
| Bladder cancer | 1.03 (0.49-2.18) |
| Colorectal cancer | 0.82 (0.53-1.27) |
| Prostate cancer | 1.18 (0.83-1.66) |
| Ovarian cancer | 0.46 (0.19-1.12) |
| Endometrial cancer | 1.02 (0.62-1.69) |
| Neuroendocrine cancer | 0.76 (0.35-1.63) |
